# A miRNA Signature for Non-Invasive Colorectal Cancer Diagnosis in Morocco: miR-21, miR-29a and miR-92a

**DOI:** 10.3390/ncrna11020026

**Published:** 2025-03-17

**Authors:** Sofia Fathi, Oussama Aazzane, Salma Guendaoui, Nezha Tawfiq, Souha Sahraoui, Fadila Guessous, Mehdi Karkouri

**Affiliations:** 1 Laboratory of Cellular and Molecular Pathology, Faculty of Medicine and Pharmacy, Hassan II University of Casablanca, Casablanca 20250, Morocco; 2 Laboratory of Pathology, Ibn Rochd University Hospital, Casablanca 20100, Morocco; 3 Mohamed VI Center for Cancer Treatment, Ibn Rochd University Hospital, Casablanca 20100, Morocco; 4 Laboratory of Oncopathology, Environment and Cancer Biology, Faculty of Medicine, Mohammed VI University of Health Sciences, Casablanca 82403, Morocco; 5 Department of Microbiology, Immunology and Cancer Biology, School of Medicine, University of Virginia, Charlottesville, VA 22908, USA

**Keywords:** miRNA, colorectal cancer, diagnosis, non-invasive, miR-21, miR-92, miR-29, Morocco

## Abstract

Colorectal cancer (CRC) is the third most diagnosed cancer and a leading cause of cancer-related mortality in Morocco, often detected at late stages. Circulating microRNAs (miRNAs) have emerged as promising non-invasive biomarkers for CRC detection, with miR-21, miR-29a, and miR-92a showing significant diagnostic potential. This study aimed to evaluate the expression levels of these miRNAs in a Moroccan population and their efficacy as diagnostic biomarkers. Methods: A prospective study was conducted using blood samples from 50 CRC patients and 50 healthy controls. Circulating miRNA expression levels were quantified through reverse transcription quantitative PCR (RT-qPCR), with normalization to miR-1228-3p. Statistical analyses, including the Mann–Whitney U test, Receiver Operating Characteristic (ROC) curve analysis, sensitivity (Sen), and specificity (Spe) evaluations, were performed to assess the diagnostic accuracy of individual miRNAs and their combined performance as panels. Results: The expression levels of miR-21, miR-29a, and miR-92a were significantly elevated in CRC patients compared to healthy controls (all *p* < 0.001). ROC analysis demonstrated that miR-92a exhibited the highest individual diagnostic performance (AUC: 0.938), followed by miR-21 (AUC: 0.907) and miR-29a (AUC: 0.898). Sensitivity and specificity were 88% and 90%, 92% and 56%, and 76% and 94%, respectively. Combinatorial analysis revealed that the miR-29a and miR-92a panel achieved the highest diagnostic accuracy (AUC: 0.976), surpassing individual miRNAs and other combinations, highlighting its potential as a robust, non-invasive biomarker panel for CRC. Conclusions: This study highlights the potential of the miR-29a and miR-92a combination, which achieved excellent diagnostic efficiency (AUC: 0.976). These findings underscore miRNA utility in enhancing early detection and reducing CRC-related mortality in Morocco.

## 1. Introduction

Colorectal cancer (CRC) represents a major public health challenge in Morocco, as highlighted by the alarming prevalence reported in the GLOBOCAN 2022 data. Specifically, CRC ranks third among the most frequently diagnosed cancers in both sexes, with over 5306 new cases identified in the year 2022, accounting for 8.3% of all diagnosed cancers in the country. Furthermore, this cancer represents a substantial mortality burden, ranking as the third leading cause of cancer-related deaths in Morocco, with 2892 attributable deaths in 2022 [1]. This high mortality rate is likely due, in part, to late diagnoses and a lack of public awareness of the disease.

Traditional procedures such as colonoscopy and sigmoidoscopy, although effective, often face patient reluctance. This highlights the urgent need for the development of non-invasive and highly accurate diagnostic tools [2]. Understanding the mechanisms of CRC pathogenesis is indeed crucial to achieving this goal. While driver gene mutations like APC (Adenomatous Polyposis Coli), TP53 (Tumor protein p53), and KRAS (Kirsten rat sarcoma viral oncogene homolog) have long been recognized as key players in the transition from adenoma to carcinoma in CRC [3], recent research suggests that microRNAs (miRNAs) may also have a critical role in this process [4].

miRNAs, short non-coding RNA molecules, play a pivotal role in post-transcriptional gene regulation by binding to 3′ untranslated regions (3′ UTRs) of the messenger RNAs (mRNAs) on target molecules and blocking their translation or degrading them [5]. Depending on their target mRNA, they can function as either oncogenes (oncomiRs) or tumor suppressor genes [6]. For instance, oncogenic miRNAs target and suppress the expression of endogenous tumor suppressor genes, thereby promoting tumorigenesis. Conversely, tumor suppressor miRNAs downregulate the expression of oncogenes associated with various cellular processes such as proliferation, apoptosis, invasion, and migration. However, due to the pleiotropic function of miRNA, their roles can be flexible, as the same miRNA can act as either a tumor suppressor or an oncogene depending on the specific tumor type or physiological conditions [7,8].

Although the vast majority of miRNAs are found within cells, a significant number are detected in the extracellular environment, circulating in biological fluids such as blood, urine, cerebrospinal fluid, tears, and saliva [9]. These circulating miRNAs are remarkably stable in these fluids due to their protection from degradation by RNases. This stability is primarily ensured through their association with Argonaute proteins, the key effector components of the miRNA-induced silencing complex (miRISC), as well as their encapsulation within microvesicles such as exosomes, microparticles, and apoptotic bodies [10,11]. Circulating miRNAs, often encapsulated within extracellular vesicles or bound to protein complexes such as Argonaute, can travel through the bloodstream to distant sites in the body. These miRNAs can be delivered to recipient cells, where they regulate gene expression and influence biological processes [12,13]. This mechanism, called intercellular communication, offers exciting new opportunities for non-invasive clinical diagnostics and prognostics.

Given the miRNAs’ regulatory role in gene expression and biological processes, their involvement in human pathology, particularly in CRC, was to be expected. Various studies have shown that there is an established link between the development of CRC and the aberrant expression of certain miRNAs. The first description was made in 2003 by Michael et al., who observed that the levels of miR-143 and miR-145 were significantly reduced in CRC tissues compared to normal tissues [14]. Since then, numerous human miRNAs have been identified as deregulated in CRC. Among them, miR-21 stands out as one of the most well-characterized and widely studied miRNAs in CRC [15]. Accumulating evidence highlights that miR-21 plays a pivotal role in the development and progression of CRC [16]. Notably, miR-21 is overexpressed in CRC cells and tissues, where it drives tumor growth, migration, and invasion by activating critical signaling pathways, including Akt, PI3K, TGF-β, and Wnt [17,18,19]. Furthermore, miR-21 has been identified as a key regulator of several target genes, including PTEN (Phosphatase and tensin homolog) a tumor suppressor gene that regulates cell growth and apoptosis, through an antagonistic relationship, where miR-21 downregulates PTEN expression, thereby promoting cell proliferation and survival of CRC cells [17,20,21]. In CRC, PTEN downregulation is frequently observed and is associated with uncontrolled activation of the PI3K/Akt pathway, leading to increased tumor cell proliferation, migration, and invasion [22]. Given its pivotal role in maintaining cellular homeostasis, PTEN loss is considered a major driver of CRC progression and metastasis.

Another emerging miRNA is miR-92a, a member of the miR-17-92 cluster, which includes five other mature miRNAs known as miR-17-5p, miR-18a, miR-19a, miR-19b-1, and miR-20a. By acting as an oncomiR in CRC, miR-92a induces cell proliferation and differentiation through the activation of oncogenic signaling pathways [23]. For instance, Zhang’s group demonstrated that the up-regulation of miR-92a negatively regulates PTEN expression in CRC, thus activating the PI3K/AKT signaling pathway, resulting in cell proliferation and lymph node metastasis [24,25,26]. In addition, studies also suggested that miR-92a regulates cell death by targeting BIM (BCL-2-interacting mediator of cell death), a protein involved in the regulation of apoptosis, thereby promoting CRC angiogenesis and proliferation [27,28].

Among the previously mentioned miRNAs exhibiting abnormal upregulation in CRC, miR-29a has emerged as a key player in the development and progression of CRC. Previous studies have revealed that the abnormal increase in miR-29a levels in CRC indicates its potential role in tumor progression. For instance, according to Tang et al., overexpressed miR-29a has been shown to significantly promote CRC metastasis by regulating MMP2/E-cad via KLF4 [29]. Another study suggested that the inhibition of miR-29a expression could elevate PTEN expression and suppress CRC cell proliferation [30]. Building upon the promising role of miRNAs in CRC diagnosis, we previously conducted a comprehensive meta-analysis involving 20 studies and 1488 CRC patients which aimed to assess the diagnostic efficacy of the most extensively studied blood-based miRNAs, namely miR-21, miR-29, and miR-92, in differentiating CRC patients from healthy controls. Our findings revealed encouraging results, demonstrating high diagnostic accuracy of the three miRNAs with a pooled sensitivity of 0.74 (CI: 0.72–0.76), a pooled specificity of 0.85 (CI: 0.83–0.87) and an area under the curve (AUC) of 0.9136 [31]. These findings underscore the potential role of these miRNAs as valuable non-invasive biomarkers for CRC diagnosis. Driven by these promising findings, we conducted a pilot study to evaluate the expression levels of circulating miR-21, miR-29a, and miR-92a as potential non-invasive diagnostic biomarkers in a Moroccan population, a demographic that has not been previously explored in this context. Our study is based on the premise that variations in the circulating levels of miR-21, miR-29a, and miR-92a can reliably distinguish CRC patients from healthy subjects, confirming their value as non-invasive diagnostic biomarkers.

## 2. Results

### 2.1. Characteristics of the Study Population

A total of 100 participants were recruited for the study, including 50 CRC patients and 50 healthy volunteers. The median age of patients was 55 (50.0–63.75 years), while that of the healthy volunteers was 52 (46.5–62.5 years). The CRC group consisted of 29 (58%) males and 21 (42%) females, whereas the control group included 18 (36%) males and 32 (64%) females. Regarding the clinical characteristics, 28 (56%) patients had tumors predominantly in the colon, while 22 (44%) patients had tumors in the rectum. Tumor laterality was predominantly observed on the left side, affecting 35 patients (70%), compared to 15 patients (30%) on the right side. The analysis of tumor size revealed that 23 (46%) CRC tumors measured ≤5 cm, while 27 (54%) were larger than 5 cm. Histopathological analysis showed that adenocarcinoma was the most common diagnosis, representing 45 patients (90%), while mucinous cell carcinoma accounted for 5 patients (10%). The majority of tumors were moderately to poorly differentiated, observed in 44 patients (88%), while a smaller proportion were well-differentiated, affecting 6 patients (12%). The analysis of microsatellite instability (MSI) status revealed that 34 (68%) CRC tumors were microsatellite stable (MSS), while 16 (32%) exhibited high microsatellite instability (MSI-H). Clinical staging revealed that 11 patients (22%) were diagnosed at stage II, 21 (42%) at stage III, and 18 (36%) at stage IV. Among patients with metastases, 15 (30%) had a single site, while 4 (8%) had two or more sites. Regarding the specific locations of metastases, 8 patients (16%) had lymph node involvement, 2 patients (4%) had lung metastases, and 14 patients (28%) presented with liver metastases. The most commonly reported symptoms among CRC patients were abdominal pain in 27 patients (54%), constipation in 36 patients (72%), and significant weight loss in 32 patients (64%). Additional symptoms included diarrhea in 13 patients (26%), bleeding per rectum in 10 patients (20%), and nausea in only 1 patient (2%). Details are provided in Table 1.

### 2.2. Comparison of miR-21, miR-92, and miR-29 Expression Levels Between CRC Patients and Healthy Volunteers

**Figure 1:**
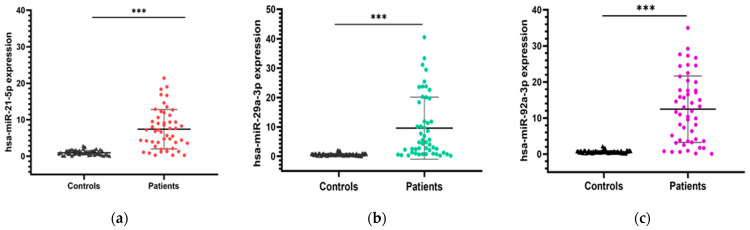
Expression levels of the three miRNA candidates in CRC patients vs. healthy controls. (**a**) miR-21 expression; (**b**) miR-29a expression; (**c**) miR-92a expression. Mann–Whitney U test was used for comparison between groups; *** *p* < 0.001.

The expression levels of the three candidate microRNAs (miR-21, miR-29a, and miR-92a) were evaluated in blood samples collected from CRC patients and healthy volunteers. To ensure accurate and reliable comparisons, normalization was performed using the endogenous control miR-1228. Our results revealed that miR-21 expression was significantly upregulated in CRC patients compared to healthy controls (7.42 ± 5.39 vs. 0.97 ± 0.64; *p* < 0.001). Similarly, the expression level of miR-29a was significantly higher in CRC patients compared to healthy individuals (9.59 ± 10.56 vs. 0.56 ± 0.38; *p* < 0.001). Additionally, miR-92a was also found to be significantly upregulated in CRC patients compared to healthy individuals (12.46 ± 9.22 vs. 0.61 ± 0.37; *p* < 0.001). Figure 1 illustrates the expression levels of the three miRNA candidates in both groups.

### 2.3. The Relationship Between Clinicopathological Characteristics of Patients with CRC and the Expression of miR-21, miR-29a and miR-92a

**Figure 2:**
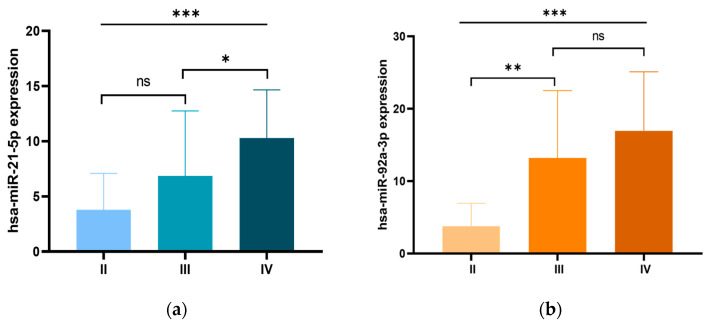
Expression levels of (**a**) miR-21 and (**b**) miR-92a in patients with CRC according to different clinical stages. ns: non-significant; * *p* < 0.05; ** *p* < 0.01; *** *p* < 0.001.

We further examined the relationship between the expression levels of the three miRNA candidates and the clinicopathological characteristics of CRC patients. Our analysis revealed a significant association between miR-21 overexpression and early-onset CRC, with significantly higher expression levels in patients diagnosed before the age of 50 compared to those diagnosed at an older age (*p* = 0.014). In addition, miR-21 overexpression was significantly associated with tumor size, with higher expression levels in tumors larger than 5 cm compared to those ≤5 cm *(p* = 0.002). Moreover, miR-21 overexpression was significantly associated with advanced clinical stages (*p* = 0.001), with particularly pronounced expression in stage IV patients compared to stage III (*p* < 0.05; Figure 2a). Higher miR-21 levels were also associated with metastatic burden, with significantly increased expression in patients with two or more metastatic sites (*p* = 0.004). Furthermore, miR-21 overexpression correlated with lymph node metastases (*p* = 0.036) and liver metastases (*p* = 0.004). Similarly, elevated miR-92a expression was significantly correlated with advanced clinical stages (*p* = 0.001), with the most notable increase occurring during the progression from stage II to stage III (*p* < 0.01). Furthermore, miR-92a expression was significantly associated with metastatic burden (*p* < 0.002), showing higher levels in patients with two or more metastatic sites, particularly in those with lymph node metastasis (*p* = 0.015), liver metastasis (*p* = 0.013). Additionally, miR-92a expression was significantly associated with a family history of CRC (*p* = 0.050), and higher CEA levels (*p* = 0.021). For miR-29a, higher expression levels were significantly associated with colon tumors (*p* = 0.004), right-sided tumors (*p* = 0.035) and tumors larger than 5 cm (*p* = 0.036). These findings are summarized in Table 1 and illustrated in Figure 2.

### 2.4. Potential Diagnostic Value of miR-21, miR-29a, and miR-92a as Biomarkers for CRC

**Figure 3:**
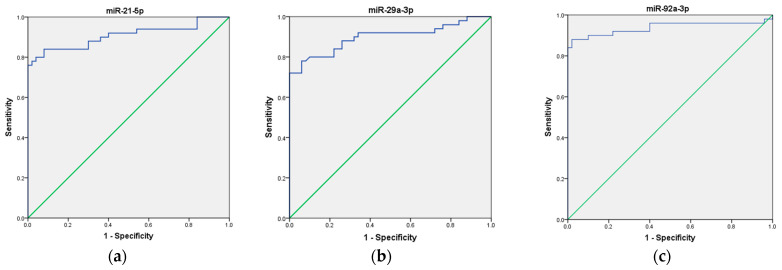
ROC curve analysis of the diagnostic potential of the individual miRNAs. (**a**) miR-21; (**b**) miR-29a; (**c**) miR-92a. The blue line represents the ROC curve, while the green diagonal line corresponds to the reference line.

To assess the potential use of these three miRNAs as a non-invasive biomarker for CRC diagnosis, we first analyzed each miRNA individually, followed by an evaluation of their combined performance as a panel. Our ROC curve analyses demonstrated that the expression of miR-21, miR-29a, and miR-92a, individually, effectively distinguished CRC patients from healthy volunteers, suggesting their potential as biomarkers in CRC. Specifically, miR-21 exhibited an AUC of 0.907 (95% CI = 0.843–0.972), indicating good overall diagnostic ability, with high sensitivity (92%), low specificity (56%), moderate positive predictive value (PPV) of 67.6%, and good negative predictive value (NPV) of 87.5%. Next, miR-29a ROC curve analysis demonstrated an AUC of 0.898 (95% CI = 0.832–0.964), indicating good overall diagnostic ability, with moderate sensitivity (76%), high specificity (94%), high PPV of 93%, and moderate NPV of 80%. Finally, miR-92a showed an AUC of 0.938 (95% CI = 0.880–0.995), indicating excellent overall diagnostic ability, with high sensitivity (88%), high specificity (90%), high PPV of 90%, and high NPV of 88%, respectively. These results are presented in Table 2 and depicted in Figure 3a–c.

Next, we evaluated the combined detection of the three candidate microRNAs (miR-21, miR-29a, and miR-92a) to determine whether their integration could enhance the diagnostic accuracy for CRC compared to the performance of each microRNA individually.

**Figure 4:**
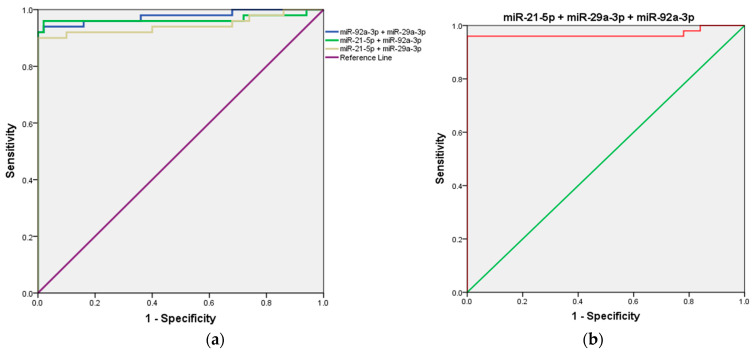
ROC curve analysis of the three-miRNA panel for CRC diagnosis. (**a**) miR-21 + miR-29a, miR-29a + miR-92a, and miR-21 + miR-92a; (**b**) miR-21 + miR-29a + and miR-92a.

First, the combined detection of miR-21 and miR-29a demonstrated a strong diagnostic performance, with an AUC of 0.944 (95% CI = 0.891–0.997; *p* < 0.001). When miR-21 was combined with miR-92a, the AUC increased further to 0.966 (95% CI = 0.920–1.000; *p* < 0.001), highlighting an enhanced diagnostic capability. Notably, the combination of miR-29a and miR-92a produced the highest AUC among all combinations, achieving 0.976 (95% CI = 0.945–1.000; *p* < 0.001), indicating superior diagnostic efficiency. Interestingly, the combined detection of all three microRNAs (miR-21, miR-29a, and miR-92a) resulted in an AUC of 0.968 (95% CI = 0.923–1.000; *p* < 0.001), which, while highly effective, did not surpass the performance of the miR-29a and miR-92a combination. Details are presented in Table 2 and Figure 4.

### 2.5. Correlation Analysis Between Circulating miR-21, miR-29a, and miR-92a

To investigate potential relationships between the circulating levels of miR-21, miR-29a, and miR-92a, a correlation analysis was performed. The results revealed a significant moderate positive correlation between miR-29a and miR-21 (*r* = 0.441, *p* = 0.001). A weak negative correlation was observed between miR-21 and miR-92a (*r* = −0.300, *p* = 0.034). However, no significant correlation was found between miR-29a and miR-92a (*r* = −0.100, *p* = 0.491). These findings suggest that miR-29a and miR-21 may be co-regulated, potentially reflecting shared biological pathways or mechanisms in CRC progression. In contrast, the lack of correlation between miR-29a and miR-92a indicates independent regulation, implying distinct functional roles in CRC.

### 2.6. In Silico Validation of miR-21, miR-92a, and miR-29a as Diagnostic Biomarkers in CRC

**Figure 5:**
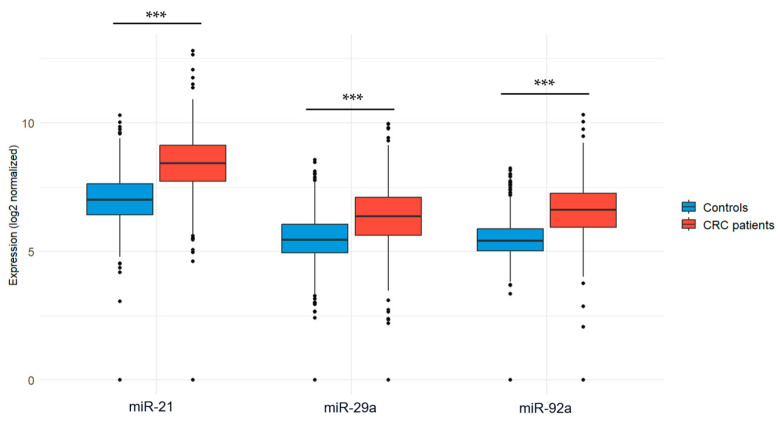
Expression levels of the 3 miRNA diagnostic biomarkers in CRC patients vs. controls using the GSE115513 dataset. Welch’s *t*-test was used for group comparisons; *** *p* < 0.001.

To further validate our findings, we performed an external validation using publicly available data from the GEO database (GSE115513). Statistical analyses confirmed a significant overexpression of miR-21, miR-92a, and miR-29a in CRC patients compared to controls, with *p*-values < 0.001 (Figure 5).

Specifically, miR-21 was highly expressed in CRC patients (8.39 ± 1.20) compared to controls (6.97 ± 1.01, *p* < 0.001). Similarly, miR-92a showed significantly elevated levels in CRC patients (6.60 ± 1.06) compared to controls (5.45 ± 0.71, *p* < 0.001), and miR-29a was also upregulated in CRC patients (6.32 ± 1.25) versus controls (5.42 ± 1.01, *p* < 0.001). The distribution of expression values is illustrated in Table 3.

**Figure 6:**
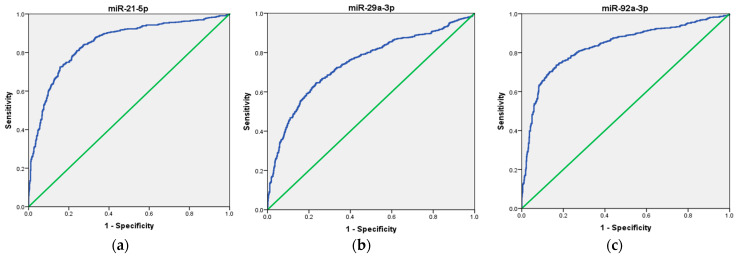
ROC curve analysis of the diagnostic potential of the individual miRNAs. (**a**) miR-21, (**b**) miR-29a, and (**c**) miR-92a in the GSE115513 dataset. The blue line represents the ROC curve, while the green diagonal line corresponds to the reference line.

Next, we assessed the diagnostic potential of these microRNAs by performing ROC curve analysis using the GSE115513 dataset. The results showed that miR-21 exhibited the highest diagnostic accuracy, with an AUC of 0.848 (95% CI: 0.827–0.868, *p* < 0.001), followed closely by miR-92a (AUC = 0.833, 95% CI: 0.811–0.855, *p* < 0.001). Although miR-29a was significantly elevated in CRC patients, it demonstrated a more moderate diagnostic performance, with an AUC of 0.746 (95% CI: 0.720–0.772, *p* < 0.001) (Figure 6). A multi-marker ROC analysis was conducted to assess whether combining these miRNAs could improve diagnostic accuracy. The simultaneous detection of miR-21, miR-92a, and miR-29a yielded a higher AUC of 0.890 (95% CI: 0.872–0.908, *p* < 0.001), suggesting that a panel-based approach may improve classification accuracy compared to individual miRNAs.

To further refine these results, we applied the Youden Index to determine the optimal cut-off values for each microRNA individually. The analysis identified 7.57 as the threshold for miR-21, achieving a sensitivity of 80.93% and a specificity of 75.96%. Likewise, miR-92a demonstrated an optimal threshold of 6.01, with a sensitivity of 73.60% and a specificity of 83.36%, while miR-29a exhibited a threshold of 7.56, yielding a sensitivity of 64.53% and a specificity of 76.58%.

**Figure 7:**
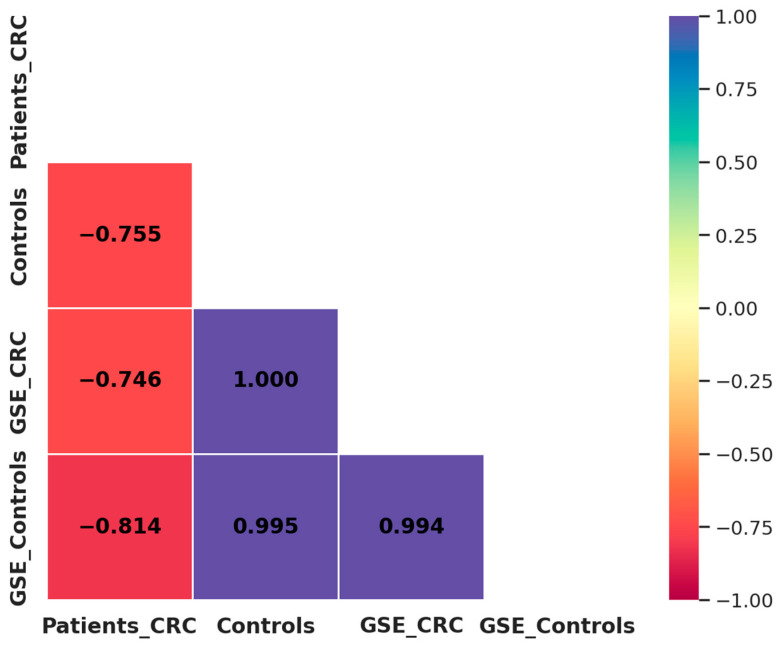
Correlation matrix of miRNA expression between our study population and the GSE115513 dataset. Patients_CRC: CRC patients from our study; Controls: healthy individuals from our study; GSE_CRC: CRC patients from the GSE115513 dataset; GSE_Controls: controls from the GSE115513 dataset.

A comparison between our results and the GSE115513 dataset revealed a strong negative correlation in miRNA expression among CRC patients (*R*^2^ = −0.746), while a highly positive correlation was observed among control individuals (*R*^2^ = 0.995) (Figure 7). These findings indicate that miRNA expression patterns in controls are highly reproducible across independent datasets, reinforcing their potential as robust biomarkers. In contrast, the observed discrepancies in CRC patients may be influenced by methodological differences. Despite these differences, the overall trend supports the diagnostic potential of miR-21, miR-29a, and miR-92a in CRC.

## 3. Materials and Methods

### 3.1. Patients Recruitment

We conducted a prospective study of 100 Moroccan participants, including 50 patients recently diagnosed with CRC at different stages and 50 healthy volunteers with no history of malignant disease. Participants were recruited consecutively from the Ibn Rochd University Hospital in Casablanca, specifically from the Mohamed VI Centre for Cancer Treatment and the Department of Visceral Surgery Emergencies, over a period from January 2022 to August 2023. The inclusion criteria were as follows: (1) newly diagnosed patients with confirmed CRC by colonoscopy and histopathology results; (2) who had not undergone surgery, chemotherapy, radiotherapy or any other treatment prior to recruitment. Exclusion criteria were as follows: (1) CRC patients harboring other synchronous malignant tumor or a history of malignant tumor; (2) CRC patients diagnosed with Crohn’s Disease or Lynch syndrome.

Clinical and pathological data, including demographics, tumor stage, location, histological subtype, and metastases, were extracted from medical records about a week after diagnosis.

### 3.2. Specimens Collection

Fresh whole blood samples (5 mL each) were collected from CRC patients a week after their diagnosis and prior to surgery or any other type of treatment, as well as from healthy volunteers for comparison. The samples were drawn in ethylene diamine tetraacetic acid (EDTA) tubes to preserve RNA integrity and were subsequently processed for total RNA extraction.

### 3.3. Total RNA Purification and microRNA Quantification

Total RNA was isolated from fresh whole blood using the QIAamp RNA Blood Mini Kit (cat. No. 52304, Qiagen, Hilden, Germany) following the manufacturer’s protocol. Initially, 1.5 mL of blood was treated with 5 volumes of Buffer EL to lyse erythrocytes, followed by leukocyte lysis with 600 μL of Buffer RLT. The resulting lysate was transferred to a QIAshredder spin column (Qiagen, Hilden, Germany) and centrifuged at maximum speed for 2 min for homogenization. To facilitate RNA binding, 600 μL of ethanol was added to the homogenized lysate, which was then transferred to the QIAamp spin column (Qiagen, Hilden, Germany). RNA selectively bound to the column membrane while impurities were removed through sequential wash steps. The purified RNA was eluted in 100 µL of RNase-free water. Finally, RNA yield and purity were quantified in duplicate using the NanoDrop 2000 spectrophotometer (Thermo Fisher Scientific, Wilmington, DE, USA) by assessing the absorbance ratios at A260/280 and A260/230. The RNA concentration extracted from plasma ranged from 3.8 to 31.3 ng/mL. All samples were divided into aliquots and stored at −80 °C until subsequent analysis.

### 3.4. cDNA Synthesis

cDNA synthesis was performed using the TaqMan Advanced miRNA cDNA Synthesis Kit (Cat. No. A28007, Thermo Fisher Scientific, Waltham, MA, USA) following the manufacturer’s instructions. Initially, miRNAs were polyadenylated by adding a poly(A) tail to the 3′ end of the mature transcript. The 5′ end was then extended through adaptor ligation. Afterward, the polyadenylated and ligated mature miRNA strands were reverse transcribed using a universal RT primer. The RT reaction was carried out using a two-step program: 15 min at 42 °C, followed by 5 min at 85 °C, using the AB 2720 thermal cycler (Thermo Scientific, Wilmington, DE, USA).

### 3.5. cDNA Preamplification

To enhance the sensitivity and reliability of miRNA candidate detection and quantification, a preamplification reaction was performed by adding miR-Amp Master Mix to 5 μL of cDNA. The PCR reaction was conducted using the AB 2720 thermal cycler (Thermo Scientific, Wilmington, DE, USA) with the following cycling conditions: an initial cycle at 95 °C for 5 min, followed by 14 cycles of 95 °C for 3 s and 60 °C for 30 s, and concluding with a stop reaction at 99 °C for 10 min. Subsequently, the cDNA samples were stored at −20 °C until they were used for quantitative Real-Time Polymerase Chain Reaction (RT-qPCR) analysis.

### 3.6. microRNAs Expression Analysis

qPCR was performed using TaqMan™ Fast Advanced Master Mix (Cat. No. 4444557, Thermo Fisher Scientific) along with specific TaqMan assays for hsa-miR-21-5p, hsa-miR-29a-3p, or hsa-miR-92a-3p (Cat. No. A25576). Each qPCR reaction mixture contained 5 μL of diluted cDNA, 1 μL of TaqMan Advanced miRNA Assays for hsa-miR-21-5p, hsa-miR-29a-3p, or hsa-miR-92a-3p, 10 μL of TaqMan Fast Advanced Master Mix (Thermo Fisher Scientific), and 4 μL of RNase-free water, yielding a final reaction volume of 20 μL. The expression levels of target miRNAs were normalized using hsa-miR-1228-3p as an endogenous control (Cat. No. A25576, Thermo Fisher Scientific), which was selected for its reported stable expression in CRC [32,33]. qPCR was conducted on the croBEE Real-Time PCR System (GeneProof, Brno, Czech Republic) to quantify the expression levels of the target miRNAs. The cycling conditions were as follows: 95 °C for 20 s, followed by 40 cycles of 95 °C for 3 s and 60 °C for 30 s. All reactions were performed in duplicate to ensure accuracy.

### 3.7. microRNAs Relative Expression

The relative expression of each candidate miRNA in CRC patients and healthy volunteers was calculated using the threshold cycle (Ct) method. Fold change was calculated using the 2^−ΔΔCt^ formula, where ΔΔCT = (Ct target miRNA − Ct miR-1228-3p) patient sample − (Ct target miRNA − Ct miR-1228-3p) healthy volunteer [34].

### 3.8. CA 19-9 CEA Analysis

Serum Carcinoembryonic antigen (CEA) and Carbohydrate antigen 19–9 (CA 19–9) were measured with a chemiluminescence immunoassay using Alinity immunoassay analyzer (Abbott Diagnostics, Chicago, IL, USA) at the time of diagnosis. CEA levels were considered normal when below 5 ng/mL, while levels ≥ 5 ng/mL were categorized as abnormal. Similarly, CA19-9 levels below 37 U/mL were classified as normal, whereas levels ≥ 37 U/mL were defined as abnormal.

### 3.9. In Silico Validation of Candidate microRNAs

To further evaluate the diagnostic potential of the identified microRNAs, we performed an external validation using publicly available data from the Gene Expression Omnibus (GEO) database. A systematic search was conducted using the following keywords: “Colorectal cancer” AND “Homo sapiens” AND “MicroRNAs” OR “miRNA”. Among the identified datasets, GSE115513 was selected for analysis, as it includes expression profiles from 750 CRC patients and 649 healthy controls, with available data for miR-21, miR-29a, and miR-92a [35]. In this dataset, total RNA was extracted from formalin-fixed paraffin-embedded (FFPE) tissue samples, and microRNA expression was quantified using the Agilent Human miRNA Microarray V19.0 platform.

### 3.10. Statistical Analysis

miRNA expression levels were quantified using the 2^−ΔΔCt^ method. The Mann–Whitney U test was applied to compare relative miRNA expression levels between the two groups. The association between clinicopathological features and the three miRNA levels was evaluated using appropriate statistical tests based on the data type (Mann–Whitney U test or Student’s *t*-test). Results are presented as mean ± standard deviation (SD). The diagnostic accuracy of candidate miRNAs and their combinations was assessed using Receiver Operating Characteristic (ROC) curve analysis with ∆Ct values, along with the calculation of the AUC. Youden’s index was then used to determine the optimal cutoff points.

Next, the expression levels of the selected miRNAs in GSE115513 were extracted and compared between CRC patients and controls using Welch’s *t*-test, which accounts for unequal variances. To ensure consistency in data processing within the external dataset (GSE115513), expression values were log-transformed (log_2_) prior to statistical analyses.

To validate our findings, a Pearson correlation analysis was performed to compare miRNA expression trends between our study and the GSE115513 dataset, assessing the reproducibility of the results.

All statistical analyses were conducted using SPSS version 22.0 (Armonk, NY, USA) and R version 4.4.2. Data visualization was performed using GraphPad Prism version 8.0 (San Diego, CA, USA) and ggplot2 in R. A *p*-value < 0.05 was considered statistically significant.

### 3.11. Ethics Approval and Consent to Participate

This study was approved by the local Ethics Committee of Ibn Rochd University Hospital in Casablanca (approval no. 02/2022) and conducted in accordance with the principles outlined in the Declaration of Helsinki. Written informed consent was obtained from all participants or their legal representatives prior to their inclusion in the study.

## 4. Discussion

In Morocco, CRC stands as the third most prevalent cancer affecting both men and women. Despite advances in medical care, the majority of CRC cases are diagnosed at advanced stages, which are frequently associated with poor prognosis, reduced quality of life, and significantly decreased survival rates. This reality underscores the urgent need for reliable, non-invasive biomarkers with strong diagnostic potential to enable earlier detection and improve patient clinical outcomes. Circulating microRNAs have emerged as key players in the tumorigenesis of various malignancies, including CRC. These small, non-coding RNA molecules, found in blood and other bodily fluids, regulate gene expression and influence critical processes such as cell proliferation, apoptosis, and metastasis. Their stability in circulation and easy detection make them highly promising candidates for non-invasive biomarkers of CRC. Based on these findings, we designed the present study to evaluate the expression of miR-21, miR-29a, and miR-92a in blood samples within a Moroccan context, aiming to provide region-specific insights into their non-invasive diagnostic potential.

Our findings reveal a significant upregulation of miR-21, miR-29a, and miR-92a in CRC patients compared to healthy controls. Notably, miR-21 expression was significantly elevated in CRC patients (7.42 ± 5.39) compared to healthy individuals (0.97 ± 0.64; *p* < 0.001). When evaluated as a non-invasive diagnostic biomarker, miR-21 demonstrated strong performance, yielding a ROC curve area of 0.907 (95% CI: 0.843–0.972) in distinguishing CRC patients from healthy subjects, with high sensitivity (92%) and low specificity (56%). These findings are consistent with those reported in previous studies, which have highlighted the significant upregulation of miR-21 in CRC patients. For example, Toiyama et al. reported that miR-21 levels were significantly elevated in the sera of CRC patients (*p* < 0.001) compared to healthy controls. Moreover, they identified miR-21 as a promising biomarker for CRC detection, demonstrating strong diagnostic performance with an AUC of 0.919 (95% CI: 0.867–0.958) [36]. These results also align with the established role of miR-21 as an oncomiR in CRC. Notably, its upregulation is significantly associated with tumor progression, actively promoting cell proliferation and metastasis [37]. Our analysis further supports this role, revealing that miR-21 overexpression is significantly associated with tumors larger than 5 cm (*p* = 0.002) and advanced clinical stage (*p* = 0.001), with levels significantly higher in stage IV patients compared to stage II (*p* < 0.001) or III (*p* < 0.05). Additionally, miR-21 overexpression was significantly linked to lymph node metastasis (*p* = 0.036) and liver metastasis (*p* = 0.004) which is consistent with the literature. For instance, Slaby et al. also demonstrated that miR-21 upregulation in CRC is significantly correlated with advanced tumor stage (*p* = 0.032) and the presence of lymph node metastases (*p* = 0.025) [38]. Interestingly, our analysis also revealed a significant association between miR-21 overexpression and early-onset CRC (<50 years, *p* = 0.014). This suggests that miR-21 may have a specific role in the development of CRC in younger patients, potentially shaping the distinct molecular profile of early-onset CRC. Given that these cases often present with more advanced disease and worse prognosis, the identification of miR-21 as a biomarker in this subgroup is particularly relevant for early detection and risk stratification. These findings underscore the clinical relevance of miR-21 not only as a diagnostic biomarker but also as a potential indicator of disease aggressiveness and metastatic potential.

Consistent with previous studies, our findings revealed a significantly higher expression of miR-29a in CRC patients (9.59 ± 10.56) compared to healthy controls (0.56 ± 0.38; *p* < 0.001). Similarly, Huang et al. reported significantly elevated miR-29a levels in plasma samples from CRC patients compared to healthy controls (*p* < 0.0001) [39]. Furthermore, the diagnostic performance of miR-29a in our study was remarkable, with an AUC of 0.898 (95% CI: 0.832–0.964), sensitivity of 76% and specificity of 94%, highlighting its potential as a reliable non-invasive biomarker for CRC detection. Similarly, a study by Liu et al. underscored the diagnostic value of serum miR-29a in CRC. Their findings revealed significantly elevated miR-29a levels in CRC patients compared to healthy controls, with an AUC of 0.878 (95% CI: 0.826–0.93). At a cut-off value of 5.020, the sensitivity was 69.4%, and the specificity was 94.9% [40]. In addition to its diagnostic performance, our findings also revealed that higher expression levels of miR-29a were significantly associated with colon tumors (*p* = 0.004) and specifically with right-sided tumors (*p* = 0.035). This association may be attributed to the distinct molecular pathways underlying the development of right-sided CRC, which are notably different from those driving left-sided CRC. In fact, right-sided tumors are frequently characterized by MSI, CpG island methylator phenotype (CIMP), and inflammation-driven carcinogenesis, all of which could influence the expression of miR-29a. In this context, miR-29a plays a significant role as a regulator, possibly linking these molecular features to tumor progression by influencing key signaling pathways. Numerous studies have demonstrated the multifaceted role of miR-29a in these processes. For example, miR-29a has been implicated in modulating immune and inflammatory pathways within the tumor microenvironment, which are often more pronounced in right-sided tumors. An investigation by Wang et al. revealed that miR-29a can activate pro-inflammatory signaling pathways such as STAT3, linking its expression to inflammation-driven carcinogenesis [41].

Beyond miR-29a, our findings also revealed a significant upregulation of miR-92a in CRC patients compared to healthy individuals (12.46 ± 9.22 vs. 0.61 ± 0.37; *p* < 0.001). This substantial difference in expression demonstrated excellent diagnostic performance, with an AUC of 0.938 (95% CI = 0.880–0.995), sensitivity of 88%, and specificity of 90%. Indeed, miR-92a’s strong diagnostic performance is consistent with prior studies. For example, Shi et al. reported that this miR-92a demonstrated high diagnostic effectiveness in CRC, with a pooled sensitivity of 81.8%, a specificity of 95.6%, and an AUC of 0.914 [42]. Building on its diagnostic potential, our study also revealed significant associations between miR-92a expression and key clinicopathological parameters. Specifically, elevated miR-92a levels were linked to lymph node metastasis (*p* = 0.015), liver metastasis (*p* = 0.013), and a family history of CRC (*p* = 0.050). These findings underscore the important role of miR-92a in CRC progression, particularly through its regulation of tumor suppressor pathways and modulation of the tumor microenvironment. Experimental studies have demonstrated a negative correlation between miR-92a and the PTEN gene, leading to activation of the PI3K/Akt signaling pathway, which promotes tumor cell migration in CRC. This evidence further suggests that miR-92a plays a critical role in facilitating lymph node metastasis in CRC patients [25]. Moreover, the observed correlation between miR-92a expression and the presence of a family history of CRC raises intriguing questions about its potential role in the genetic predisposition. While the role of miR-92a in familial CRC remains underexplored, its known regulatory influence on critical genetic pathways, such as Wnt/β-catenin signaling, provides a plausible link to hereditary tumor development. The Wnt/β-catenin pathway is frequently dysregulated in hereditary CRC syndromes, such as familial adenomatous polyposis (FAP) and Lynch syndrome [43], suggesting that miR-92a may contribute to these inherited conditions by modulating pathway components. In addition to these mechanisms, recent studies have identified miR-92a as a key player in colorectal cancer stem cells (CSCs) [44]. Viswanathan et al. demonstrated that miR-92a is selectively expressed in normal colonic stem cells and is aberrantly upregulated in CSCs isolated from CRC tissues. Importantly, miR-92a was found to regulate the tumor suppressor gene LRIG1, which plays a crucial role in inhibiting EGFR signaling and maintaining stem cell homeostasis. Increased miR-92a expression diminishes the tumor suppressive function of LRIG1 in CSCs, potentially driving tumor initiation and progression [45]. Given that CSCs are implicated in both tumorigenesis and metastasis, this mechanism may partially explain the observed association between miR-92a, metastatic burden, and family history of CRC. These findings underscore the multifaceted role of miR-92a in CRC pathogenesis, not only as a diagnostic biomarker but also as a contributor to metastatic behavior and potentially inherited disease risk. Future studies should focus on elucidating the molecular mechanisms linking miR-92a to these clinicopathological parameters and its usage in personalized CRC management.

Based on the results of individual diagnostic performances, we investigated whether combining these microRNAs could further enhance the accuracy of CRC diagnosis. Our results showed that combined detection of miR-29a and miR-92a achieved the highest AUC of 0.976 (95% CI: 0.945–1.000; *p* < 0.001), reflecting superior diagnostic efficiency, compared to the inclusion of all three microRNAs, which resulted in a slightly lower AUC of 0.968 (95% CI: 0.923–1.000; *p* < 0.001). This result suggests that specific combinations, such as miR-29a and miR-92a, may better capture synergistic effects, leading to enhanced diagnostic accuracy.

To further investigate the relationships between these circulating miRNAs, we performed a correlation analysis. The results revealed a moderate positive correlation between miR-29a and miR-21 (*r* = 0.441, *p* = 0.001), suggesting potential co-regulation of these two miRNAs, possibly reflecting shared biological pathways involved in CRC progression. The significant association between these miRNAs aligns with previous evidence indicating that both miR-21 and miR-29a are involved in tumor cell proliferation, invasion, and resistance to apoptosis.

Next, we accessed publicly available data from the GEO database (GSE115513) to externally validate our findings and reinforce their reliability. Statistical analyses confirmed a significant overexpression of miR-21, miR-29a, and miR-92a in CRC patients compared to healthy controls (*p* < 0.001), reinforcing the robustness of our results. Moreover, ROC curve analysis in the external dataset demonstrated strong diagnostic performances, with AUC values of 0.848 for miR-21, 0.833 for miR-92a, and 0.746 for miR-29a, further supporting their potential as reliable diagnostic biomarkers for CRC.

To further investigate the consistency of miRNA expression patterns between our study and the GSE115513 dataset, we performed a Pearson correlation analysis. A strong correlation was observed for control samples (*R*^2^ = 0.995, *p* = 0.061), indicating that miRNA expression remains stable across independent datasets in controls. However, a moderate negative correlation was noted in CRC patients (*R*^2^ = −0.746, *p* = 0.464), suggesting greater variability in tumor-derived miRNA expression. This discrepancy may be influenced by methodological differences (qRT-PCR vs. microarray), sample type variations (blood vs. FFPE tissues), and biological heterogeneity among CRC patients. Additionally, differences in tumor microenvironment dynamics and exosomal miRNA release could contribute to variations in circulating miRNA profiles, as tumor-derived miRNAs may not fully reflect those found in tissue samples. Furthermore, patient heterogeneity, including variations in tumor stage, genetic background may also impact miRNA expression, leading to differences across datasets. Despite these variations, the consistent overexpression of miR-21, miR-29a, and miR-92a in both studies reinforces their diagnostic relevance in CRC.

Our study is the first to evaluate the combined diagnostic performance of miR-21, miR-29a, and miR-92a for CRC detection, making it a novel contribution to the field. While numerous studies have demonstrated the individual diagnostic roles of circulating miR-21, miR-29, and miR-92 in CRC, none have explored their combined effect in enhancing diagnostic accuracy. Furthermore, it is the first to assess this combination within a North African context, addressing a significant gap in regional research. While the results are highly promising, several limitations of this study must be acknowledged. First, the sample size was relatively modest, which may restrict the generalizability of the findings to larger and more diverse populations. Second, the study was conducted within a North African context. While this represents a strength in addressing underrepresented populations, the results may not be entirely applicable to other geographic or ethnic groups due to potential differences in genetic, environmental, and dietary factors that influence microRNA expression. Moreover, the findings of this study require external validation in larger, independent cohorts to confirm their robustness and clinical relevance. Finally, future studies should also incorporate analyses that clarify the mechanisms of miRNA release in CRC, specifically, by investigating whether these miRNAs originate from tumor-derived extracellular vesicles or other circulating entities. Such investigations would provide deeper insights into their biological origins and could enhance the specificity of our findings.

## 5. Conclusions

This study underscores the diagnostic potential of miR-21, miR-29a, and miR-92a in CRC, with the combination of miR-29a and miR-92a (sensitivity: 82%, specificity: 92%) emerging as the most effective for diagnosis. In particular, this combination shows promise for preventive screening in individuals at higher risk, such as those with a family history or genetic predispositions. These findings were further supported by an in silico validation using the GSE115513 dataset, reinforcing the diagnostic relevance of these miRNAs. However, additional validation in larger and more diverse cohorts remains necessary to confirm their clinical applicability as non-invasive biomarkers for CRC.

## Figures and Tables

**Table 1:** Association between clinicopathological characteristics and miRNA expression in CRC patients.

Category	Patients	miR-21 Expression		miR-29a Expression		miR-92a Expression	
	N (%)	Mean ± SD	*p*-Value	Mean ± SD	*p*-Value	Mean ± SD	*p*-Value
Sex							
Male	29 (58%)	7.58 ± 5.14	0.630	10.93 ± 12.07	0.945	12.81 ± 10.11	0.867
Female	21 (42%)	7.19 ± 5.84		7.74 ± 7.95		11.98 ± 8.05	
Age at diagnosis, years							
<50	10 (20%)	8.25 ± 0.96	**0.014**	8.51 ± 3.48	0.395	11.48 ± 1.46	0.102
≥50	40 (80%)	3.50 ± 1.25		10.53 ± 1.66		16.40 ± 2.62	
Family history of CRC							
Yes	38 (76%)	9.38 ± 6.02	0.180	11.83 ± 13.70	0.874	17.37 ± 9.92	**0.050**
No	12 (24%)	6.80 ± 5.11		8.88 ± 9.47		10.92 ± 8.55	
Smoking status							
Yes	16 (32%)	7.87 ± 5.04	0.205	7.80 ± 8.44	0.460	9.57 ± 8.59	0.134
No	34 (68%)	6.46 ± 6.14		10.43 ± 11.44		13.83 ± 9.31	
ECOG PS							
0	31 (62%)	7.36 ± 5.08	0.960	9.56 ± 10.33	0.849	13.38 ± 9.34	0.303
1	19 (38%)	7.51 ± 6.01		9.63 ± 11.22		10.97 ± 9.06	
Tumor location							
Colon	28 (56%)	7.63 ± 5.56	0.769	12.30 ± 11.02	**0.004**	12.14 ± 8.84	0.769
Rectum	22 (44%)	7.14 ± 5.28		6.13 ± 9.04		12.87 ± 9.88	
Tumor sidedness							
Right	15 (30%)	9.40 ± 6.17	0.122	12.74 ± 9.68	**0.035**	13.68 ± 10.75	0.695
Left	35 (70%)	6.57 ± 4.87		8.24 ± 10.76		11.94 ± 8.60	
Tumor size, cm							
≤5	23 (46%)	4.27 ± 0.78	**0.002**	6.96 ± 1.93	**0.036**	12.43 ± 2.24	0.741
>5	27 (54%)	9.88 ± 1.24		12.82 ± 2.11		12.49 ± 1.51	
Histological type							
Adenocarcinoma	45 (90%)	6.90 ± 4.93	0.132	9.21 ± 10.80	0.159	12.73 ± 9.58	0.638
Mucinous cell carcinoma	5 (10%)	12.08 ± 7.62		12.99 ± 8.15		10.04 ± 4.86	
Histological grade							
Well-differentiated	6 (12%)	7.54 ± 6.20	1	13.65 ± 6.98	0.070	10.49 ± 9.00	0.694
Moderately to poorly differentiated	44 (88%)	7.40 ± 5.35		9.04 ± 10.90		12.73 ± 9.32	
MSI Status							
MSS	34 (68%)	8.03 ± 1.01	0.129	9.30 ± 1.67	0.610	11.55 ± 1.55	0.270
MSI-H	16 (32%)	5.74 ± 1.54		11.88 ± 3.06		14.41 ± 2.39	
Clinical stage							
II	11(22%)	3.78 ± 3.30	**0.001**	9.77 ± 8.06	0.844	3.78 ± 3.15	**0.001**
III	21 (42%)	6.86 ± 5.88		9.40 ± 10.45		13.18 ± 9.31	
IV	18 (36%)	10.29 ± 4.36		9.70 ± 12.43		16.93 ± 8.17	
Metastatic burden							
0	31 (62%)	5.85 ± 5.38	**0.004**	9.45 ± 9.71	0.979	9.47 ± 8.65	**0.002**
1	15 (30%)	9.29 ± 4.49		10.50 ± 13.05		14.64 ± 6.32	
≥2	4 (8%)	12.54 ± 3.67		7.23 ± 8.45		27.48 ± 6.14	
Lymph node metastasis							
Yes	8 (16%)	10.51 ± 4.01	**0.036**	9.88 ± 10.91	0.649	20.00 ± 9.34	**0.015**
No	42 (84%)	6.38 ± 5.46		8.04 ± 8.93		11.03 ± 8.57	
Lung metastasis							
Yes	2 (4%)	9.25 ± 0.36	0.392	16.01 ± 21.36	0.720	24.02 ± 5.14	0.069
No	48 (96%)	7.34 ± 5.49		9.32 ± 10.23		11.98 ± 9.06	
Liver metastasis							
Yes	14 (28%)	10.60 ± 4.83	**0.004**	8.55 ± 11.82	0.674	17.75 ± 8.46	**0.013**
No	36 (72%)	6.18 ± 5.14		9.99 ± 10.18		10.41 ± 8.77	
Abdominal pain							
No	23 (46%)	7.52 ± 4.71	0.668	7.79 ± 7.72	0.984	12.50 ± 9.47	0.946
Yes	27 (54%)	7.33 ± 6.00		11.12 ± 12.43		12.43 ± 9.19	
Nausea							
Yes	1 (20%)	12.29	0.400	0.72	0.320	3.63	0.520
No	49 (80%)	7.32 ± 5.40		9.77 ± 10.59		12.65 ± 9.23	
Constipation							
Yes	36 (72%)	7.83 ± 5.55	0.430	9.34 ± 9.85	0.966	12.20 ± 8.49	0.897
No	14 (28%)	6.36 ± 5.00		10.24 ± 12.58		13.15 ± 11.22	
Diarrhea							
Yes	13 (26%)	9.15 ± 6.31	0.181	11.95 ± 13.05	0.781	12.06 ± 11.05	0.699
No	37 (74%)	6.81 ± 4.98		8.76 ± 9.61		12.61 ± 8.66	
Bleeding per rectum							
Yes	10 (20%)	8.03 ± 5.45	0.097	4.37 ± 3.40	0.274	10.54 ± 7.45	0.624
No	40 (80%)	4.94 ± 4.60		10.89 ± 11.35		12.95 ± 9.63	
Significant weight loss							
Yes	32 (64%)	8.14 ± 5.76	0.253	8.83 ± 9.31	0.903	13.30 ± 9.93	0.518
No	18 (36%)	6.13 ± 4.52		10.95 ± 12.66		10.99 ± 8.15	
CEA level (ng/mL)							
<5	34 (68%)	7.53 ± 5.12	0.755	10.70 ± 11. 27	0.519	10.37 ± 8.87	**0.021**
≥5	16 (32%)	7.17 ± 6.09		7.22 ± 8.73		16.93 ± 8.56	
CA19-9 (U/mL)							
>37	41 (82%)	7.33 ± 5.27	0.830	9.61 ± 10.49	0.890	11.79 ± 8.84	0.357
≤37	9 (18%)	7.82 ± 6.22		9.51 ± 11.54		15.56 ± 10.80	

**Table 2:** Diagnostic performance of miR-21, miR-29a, and miR-92a individually and in combination as panels.

miRNA	SEN (%)	SPE (%)	PPV (%)	NPV (%)	AUC (95% CI)	*p*-Value
miR-21	92	56	67	87	0.907 (0.843–0.972)	<0.001
miR-29a	76	94	93	80	0.898 (0.832–0.964)	<0.001
miR-92a	88	90	90	88	0.938 (0.880–0.995)	<0.001
miR-21 + miR-29a	84	75	77	82	0.944 (0.891–0.9970	<0.001
miR-21 + miR-92a	90	73	77	88	0.966 (0.920–1.000)	<0.001
miR-29a + miR-92a	82	92	92	84	0.976 (0.954–1.000)	<0.001
miR-21 + miR-29a + miR-92a	85	80	81	84	0.968 (0.923–1.000)	<0.001

**Table 3:** Differential expression of miR-21, miR-92a, and miR-29a in CRC patients versus controls in GSE115513 dataset.

miRNA Expression	CRC Patients	Healthy Volunteers	*p*-Value
	Mean ± SD	Mean ± SD	
miR-21	8.39 ± 1.20	6.97 ± 1.01	<0.001
miR-29a	6.32 ± 1.25	5.42 ± 1.01	<0.001
miR-92a	6.60 ± 1.06	5.45 ± 0.71	<0.001

## Data Availability

Data supporting the findings of this study are available upon reasonable request from the corresponding author.

## Abbreviations

The following abbreviations are used in this manuscript:

CRC	Colorectal Cancer
APC	Adenomatous Polyposis Coli
KRAS	Kirsten Rat Sarcoma Viral Oncogene Homolog
miRNAs	MicroRNAs
mRNAs	Messenger RNAs
miRISC	miRNA-Induced Silencing Complex
PTEN	Phosphatase and Tensin Homolog
BIM	Bcl-2-Like Protein 11
AUC	Area Under the Curve
EDTA	Ethylenediaminetetraacetic Acid
Ct	Cycle threshold
CEA	Carcinoembryonic Antigen
MSI	Microsatellite instability
MSS	Microsatellite stable
MSI-H	High microsatellite instability
CA 19-9	Carbohydrate Antigen 19-9
SD	Standard Deviation
ROC	Receiver Operating Characteristic
PPV	Positive Predictive Value
NPV	Negative Predictive Value
